# Examining the Associations Between Menstrual Disorders and SARS-CoV-2 Exposure in Greek Adolescents: A Retrospective Cohort Study

**DOI:** 10.3390/jcm15208033

**Published:** 2026-10-16

**Authors:** Asimenia Svigkou, Charikleia Stefanaki, Eleni Kokkou, Dimitrios Kalogirou, Vasiliki Efthymiou, Stavroula Papadodima, Flora Bacopoulou

**Affiliations:** 1 Center for Adolescent Medicine and UNESCO Chair in Adolescent Health Care, First Department of Pediatrics, School of Medicine, National and Kapodistrian University of Athens, Aghia Sophia Children’s Hospital, 11527 Athens, Greece; 2 Unit of Developmental and Behavioral Pediatrics, First Department of Pediatrics, National and Kapodistrian University of Athens, Aghia Sophia Children’s Hospital, 11527 Athens, Greece; 3 Department of Public and Community Health, School of Public Health, University of West Attica, 11521 Athens, Greece; pch23679083@uniwa.gr; 4 School of Medicine, European University Cyprus, 1516 Nicosia, Cyprus; 5 Department of Forensic Medicine and Toxicology, School of Medicine, National and Kapodistrian University of Athens, 10679 Athens, Greece; stpapd@med.uoa.gr

**Keywords:** adolescence, heavy menstrual bleeding, menstruation, abnormal uterine bleeding, SARS-CoV-2, COVID-19, coronavirus disease 2019, menstrual disorders, coronavirus, severe acute respiratory syndrome coronavirus 2

## Abstract

**Background**: Exposure to severe acute respiratory syndrome coronavirus 2 (SARS-CoV-2), whether *via* infection or vaccination, seems to influence the character of the menstrual cycle by disrupting hormonal and immune function. **Methods**: The main aim of this cohort study was to explore the association between exposure to SARS-CoV-2—through either vaccination or infection—and menstrual disorders in adolescent females. We also aimed at identifying the most prevalent menstrual disorders observed and assessing potential differences between the exposure groups that may contribute to these conditions. **Results**: This study included data from adolescent females who visited the Center for Adolescent Medicine and UNESCO Chair in Adolescent Health Care due to various menstrual disorders. Data from 92 adolescents, 77 participants exposed to SARS-CoV-2 and 15 controls, were retrieved. Significant associations were found between exposure to SARS-CoV-2 and abnormally frequent uterine bleeding (*p* = 0.032) and abnormal uterine bleeding (*p* = 0.025). Adolescents with a past confirmed case of coronavirus disease 2019 (COVID-19) demonstrated significantly lower serum concentrations of fibrinogen (*p* = 0.023) and iron (*p* = 0.03), as well as higher γ-GT concentrations (*p* = 0.028), than those who were non-infected, whereas vaccinated adolescents presented with significantly lower concentrations of coagulation factors VIII (*p* = 0.002), vWFAg (*p* = 0.023), and Rcof (*p* = 0.007) than those of their unvaccinated peers. Overall, adolescents exposed [infection (or/and) vaccination] to SARS-CoV-2 demonstrated increased estradiol (E2) concentrations compared to those of the controls. In conclusion, adolescent exposure to SARS-CoV-2 was strongly linked to abnormal uterine bleeding and polymenorrhea, potentially due to decreased coagulation factors or increased E2 concentrations. **Conclusions**: Further research involving larger samples and a more sophisticated design is needed to validate these findings.

## 1. Introduction

The menstrual cycle serves as an important indicator of female health, regulated by the hypothalamic–pituitary–gonadal (HPG) axis. In adolescents, typical cycle length ranges from 21 to 45 days, with an average of 32.2 days during the first two years after menarche. Early cycles are often anovulatory, reflecting the immaturity of the HPG axis. Menstrual bleeding usually lasts between 3 to 7 days, with adolescents using approximately 3 to 6 sanitary products per day [1,2]. The HPG axis functions through endocrine feedback loops, in which sex steroid hormones regulate the secretion of gonadotropin-releasing hormone (GnRH), luteinizing hormone (LH), and follicle stimulating hormone (FSH) [3]. Disruptions within this system can result in menstrual irregularities [1].

Menstrual disorders are common among adolescents, often appearing around the time of menarche. These disorders are typically defined by abnormalities in the frequency, duration, or intensity of menstrual bleeding [4].

Emerging data suggest that exposure to severe acute respiratory syndrome coronavirus 2 (SARS-CoV-2), whether *via* infection [coronavirus disease 2019 (COVID-19)] or vaccination, may influence menstrual patterns by disrupting hormonal and immune function [5,6,7,8]. SARS-CoV-2 binds to angiotensin-converting enzyme 2 (ACE2) receptors, which are expressed in ovarian and endometrial tissues and are part of the renin–angiotensin system (RAS) [6]. Disruption of the RAS can interfere with ovulation, ovarian hormone production and endometrial function, potentially leading to menstrual irregularities [9,10,11]. Angiotensin II, a key molecule within the RAS, plays a critical role in reproductive health, and its imbalance may contribute to abnormal uterine bleeding [11].

Pandemic-related stress may also contribute indirectly to menstrual disturbances [8]. Stress activates the hypothalamic–pituitary–adrenal (HPA) axis, leading to increased cortisol concentrations, suppressing the release of GnRH. This suppression reduces the downstream secretion of FSH, LH, and estrogen [12], possibly resulting in anovulatory cycles and various menstrual disorders [12,13,14]. Furthermore, cortisol can directly impair the reproductive function by affecting the ovarian granulosa and luteal cells [15]. Immune activation following SARS-CoV-2 exposure may also influence menstrual health. Dysregulated immune responses can stimulate the HPA axis, which in turn suppresses the HPG axis [16,17,18,19]. Inflammatory cytokines have been shown to disrupt ovarian functions, including ovulation [20]. Additionally, alterations in immune cell composition within the endometrium may interfere with normal shedding and repair, contributing to abnormal uterine bleeding [7,21,22]. Immune-mediated coagulopathies have been reported in the context of SARS-Cov-2, potentially leading to either bleeding or thrombosis [8,23].

SARS-CoV-2 exposure may also lead to increased prolactin concentrations, which can interfere with GnRH secretion and consequently disrupt downstream reproductive hormones [24,25]. Hyperprolactinemia may inhibit ovulation and cause menstrual irregularities [26,27].

Numerous studies have reported statistically significant relationships among vaccination against SARS-CoV-2, or COVID-19, and mild, temporary menstrual changes, usually resolving within months [28,29,30,31,32,33]. However, most of these studies have focused on adult females, with limited data available on adolescents [34,35,36]. To date, no similar study has been conducted in Greece.

This study aimed to examine the possible association between SARS-CoV-2 exposure, either *via* infection or vaccination, and the occurrence of menstrual disorders in Greek adolescent girls. Additionally, this study seeks to characterize the most prevalent menstrual irregularities within this population and to assess potential differences in menstrual patterns between adolescents who were exposed to SARS-CoV-2 and those who were not.

## 2. Materials and Methods

### 2.1. Study Design—Setting

This retrospective cohort study was conducted at the Center for Adolescent Medicine and UNESCO Chair in Adolescent Health Care, Choremeion Research Laboratory of the First Department of Pediatrics, School of Medicine, National and Kapodistrian University of Athens, Aghia Sophia Children’s Hospital. The study period spanned from 4 January 2020, to 31 December 2024. Ethical approval was obtained from the Ethics Committee of the Aghia Sophia Children’s Hospital, ensuring full adherence to medical ethical standards and the protection of participants’ rights and anonymity (Protocol Number: 26068/22.10.2024).

### 2.2. Participants, Data Sources, and Variables

Data deemed relevant to the objectives of the study were retrieved from the medical records of adolescents. Elements of the clinical examinations and the results of laboratory samples obtained on the 3rd to 5th days of the menstrual cycle were recorded. The study sample consisted of adolescent females, aged 12 to 17 years, who presented to the Center for Adolescent Medicine with reported menstrual irregularities 2 to 6 months after exposure to the SARS-CoV-2. To enhance the internal validity of the study, stringent inclusion criteria were applied. Participants were eligible if they had attained menarche at least two years prior to confirmed SARS-CoV-2 exposure—either through infection or vaccination—and had no documented medical conditions known to be associated with amenorrhea or menorrhagia. The diagnosis of SARS-CoV-2 infection was established through either rapid antigen testing or molecular confirmation via reverse transcription polymerase chain reaction (RT-PCR). To minimize the confounding effects of physiological immaturity of the HPG axis, exclusion criteria included adolescents with primary amenorrhea or those whose menarche occurred within two years of SARS-CoV-2 exposure. Specifically, information collected included the chronological age of participants, age at menarche, and exposure to SARS-CoV-2, either *via* vaccination or infection, ensuring that at least two years since menarche had elapsed at the time of exposure. Gynecological history was also documented, including menstrual cycle length, duration of menstruation, and the average daily use of sanitary products, to assess the characteristics of the menstrual cycle and to identify menstrual disorders. Additionally, laboratory test results were recorded to explore possible differences between adolescents exposed to SARS-CoV-2 and controls.

### 2.3. Definitions

Abnormal uterine bleeding (AUB) was defined as bleeding originating from the uterine corpus that was abnormal in frequency, regularity, duration, and/or volume. In adolescents, menstrual cycles occurring at intervals of <21 days or >45 days, menstrual bleeding lasting >7 days, or excessive menstrual blood loss were considered abnormal. Secondary amenorrhea in adolescents was defined as the absence of menstruation for more than 3 months (90 days) in an individual who has previously menstruated. In adolescents, an interval of more than 90 days between menstrual periods is considered uncommon and warrants clinical evaluation [37]. Abnormally frequent menstrual cycles occurring more frequently than every 21 days are defined as abnormally frequent menstrual cycles. Oligomenorrhea refers to menstrual cycles occurring at intervals longer than 45 days. Heavy menstrual bleeding (HMB), historically termed menorrhagia, is defined as menstrual bleeding that adversely affects physical, social, emotional, or material quality of life. In adolescents, bleeding lasting more than 7 days or requiring menstrual products to be changed every 1–2 h is suggestive of excessive menstrual blood loss [4].

Exposure in this study was defined as either receiving at least one dose of a SARS-CoV-2 vaccine or a laboratory-confirmed diagnosis of COVID-19. The control group consisted of participants who repeatedly tested negative for SARS-CoV-2 and who had remained asymptomatic of infection for at least 6 months before the study. The primary outcome was the presence of menstrual disorders, evaluated across three key parameters: (1) alterations in the menstrual cycle length, (2) changes in the duration of menstrual bleeding, measured in days, and (3) variations in the menstrual blood volume. Menstrual volume was indirectly estimated *via* self-reported data on the average number of sanitary products (pads or tampons) used per day during menstruation. 

### 2.4. Statistical Analysis

A two-sided statistical *p*  <  0.05 was established as the threshold for statistical significance. Missing data, where relevant information was unavailable in the medical records, were excluded from the analysis to preserve data integrity. All statistical analyses were performed using IBM SPSS Statistics for Windows, version 29.0 (IBM Corp., Armonk, NY, USA). Descriptive statistics were used to characterize the study population. Categorical variables were summarized using absolute frequencies (*n*) and relative frequencies (%), while quantitative variables were reported as means. Data related to menstrual cycle characteristics—specifically, cycle length, duration of menstruation, and frequency of sanitary product usage—were recoded into categorical variables representing specific types of menstrual irregularities. To further explore differences in biological markers, the Student’s *t*-test was applied to compare laboratory test results across study groups: (1) vaccinated vs. unvaccinated, (2) infected vs. non-infected, and (3) SARS-CoV-2-exposed vs. control participants. Associations between categorical variables were assessed using the Chi-square (χ^2^) test, with Fisher’s exact test applied when the expected cell frequencies were insufficient for the Chi-square test. In all analyses, *p*-values and corresponding 95% confidence intervals were reported to determine statistical significance and assess the precision of the estimates.

## 3. Results

### 3.1. Participants

A total of 92 adolescent females diagnosed with menstrual disorders and meeting the predefined inclusion criteria were enrolled in this retrospective cohort study. Of these, 77 participants (83.7%) had documented exposure to SARS-CoV-2, either through vaccination or laboratory-confirmed infection, while the remaining 15 participants (16.3%) constituted the unexposed control group. Among the exposed individuals, 37 adolescents (40.2%) had received at least one dose of a SARS-CoV-2 vaccine, and 69 (75.0%) had experienced a confirmed COVID-19 infection. Only one statistically significant difference in estradiol concentrations (*p* < 0.001) was observed between the groups. In adolescents who had been exposed to SARS-CoV-2, the mean estradiol concentration was 53.20 pg/mL, while in those who had not been exposed, it was 27.67 pg/mL (Table 1).

Secondary amenorrhea was observed in 23 adolescents (25%). Regarding the menstrual cycle length, 18 participants (19.6%) experienced oligomenorrhea, while 13 (16.3%) had abnormally frequent menstrual cycles. Abnormal uterine bleeding was observed in 22 adolescents (25%), while HMB was reported in 47 adolescents (52.2%). Regarding menstrual flow, 24 participants (28.2%) presented with HMB, while 32 adolescents (36%) experienced menstruation lasting longer than 7 days. Statistically significant associations were found between SARS-CoV-2 exposure (vaccination or/and infection) and abnormally frequent menstrual bleeding (χ^2^ = 4.765; df = 1; *p* = 0.043), as well as between SARS-CoV-2 exposure and duration of menstrual cycle (χ^2^ = 6.063; df = 2; *p* = 0.048).

### 3.2. Comparisons Between Adolescents Vaccinated and Unvaccinated Against SARS-CoV-2

Statistically significant differences were identified regarding coagulation factors VIII (*p* = 0.002), vWFAg (*p* = 0.023), and Rcof (*p* = 0.007). Vaccinated adolescents presented with lower concentrations of these factors than those of their unvaccinated peers. The mean concentrations among vaccinated adolescents were 86.3% for factor VIII, 71.84% for vWFAg, and 65.5% for Rcof vs. 136.25%, 108.54%, and 110.83% in unvaccinated adolescents, respectively (Table 2).

### 3.3. Comparison Between COVID-19 Infected and Non-Infected Adolescents

In adolescents with a past confirmed COVID-19 infection (vs. non-infected), the concentrations of fibrinogen (*p* = 0.023) and iron (*p* = 0.03) were reduced, while the concentrations of γ-GT were increased (*p* = 0.028). More specifically, in the infected adolescents, mean fibrinogen concentration was 257.26 mg%, mean iron concentration was 62.43 μg/dL, and mean γ-GT concentration was 13.86 U/L vs. 312.31 mg%, 88 μg/dL, and 10.04 U/L in the uninfected adolescents, respectively (Table 3).

## 4. Discussion

The findings of this study demonstrate a statistically significant association of SARS-CoV-2 exposure with abnormal frequency and duration of menstrual cycles. Analysis of laboratory parameters revealed reduced levels of coagulation factor VIII, von Willebrand factor antigen (vWF:Ag), and ristocetin cofactor activity (RCoF) in vaccinated adolescents, whereas lower fibrinogen levels were observed among those with a history of COVID-19 infection. These findings suggest that both SARS-CoV-2 infection and vaccination may be associated with subtle alterations in the hemostatic system. Additionally, adolescents exposed to SARS-CoV-2 exhibited higher estradiol concentrations, a finding of potential clinical relevance given that hormonal dysregulation may disrupt menstrual cycle homeostasis and contribute to the development of menstrual abnormalities. Notably, although statistically significant differences were observed between groups, all laboratory parameters remained within their respective physiological reference ranges. Both COVID-19 and SARS-CoV-2 vaccination have been associated with coagulopathies, which may manifest either as bleeding and thrombocytopenia or as increased coagulation and thrombus formation [38,39]. Although thrombotic events are the most common complications following exposure to SARS-CoV-2, hemorrhagic episodes can also be common. Several factors contribute to the greater predisposition to bleeding in COVID-19 patients, including thrombocytopenia, consumption of coagulation factors, hyperactivation of the fibrinolytic system, low iron concentrations and the administration of anticoagulants for thromboprophylaxis. The simultaneous activation of coagulation and fibrinolytic systems, often triggered by pro-inflammatory cytokines, leads to the consumption of clotting factors, resulting in thrombotic and hemorrhagic events [40].

The coagulation system plays an essential role in the regulation of menstruation [41]. Findings from the study indicate that exposure to SARS-CoV-2 is linked to reduced concentrations of coagulation factors, which may contribute to the onset of menstrual disorders such as abnormally frequent menstrual cycles and HMB. Abnormal menstrual bleeding is a frequent issue among adolescents. While approximately 95% of cases are attributed to anovulatory cycles due to the immaturity of the HPG axis, other underlying pathologies may also be present. Among these, systemic disorders affecting hemostasis are increasingly recognized as a common cause of menstrual irregularities in adolescent girls [38,42].

Furthermore, menstrual disorders are closely linked to abnormal concentrations of estrogen and progesterone. Estradiol mainly regulates the length of the menstrual cycle and secondarily affects the intensity and duration of bleeding [43]. It predominates the follicular phase, while progesterone is dominant during the luteal phase, which typically lasts about 14 days. Variations in the follicular phase largely determine the overall cycle length [2].

Previous research has shown that elevated concentrations of estradiol and FSH during the follicular phase are associated with shorter menstrual cycle lengths, while longer cycles are linked to higher concentrations of LH and lower concentrations of estradiol in the same phase. The same study also found irregularities in cycle length, connected to an increased risk of anovulatory cycles [44]. Therefore, the increased estradiol concentrations observed in adolescents exposed to SARS-CoV-2 may contribute to a higher risk of menstrual disorders such as abnormal uterine bleeding and polymenorrhea. However, the precise biological mechanisms linking hormonal fluctuations to menstrual cycle length remain unclear and require further research for clarification.

Several studies involving adolescents have indicated that SARS-CoV-2 exposure is associated with menstrual disorders. Mohr-Sasson et al. reported that among 22 out of 35 girls with previously regular menstruation, 7 (31.8%) experienced irregular menstrual patterns after receiving the vaccine [35]. Caspersen et al. noticed an increased risk of menstrual disorders in the first cycle following a single dose of the COVID-19 vaccine. More specifically, the likelihood of experiencing heavier or prolonged bleeding was significantly higher compared to menses before vaccination. Additionally, there was increased risk of shorter or longer cycle intervals and increased menstrual pain after vaccination [34]. Aldali et al. found that adolescent girls with chronic illness had 2.4 times higher odds of experiencing menstrual irregularities post-vaccination compared to those without chronic conditions [36].

The lack of a power analysis to determine the necessary sample size, which may affect the reliability and generalization of the findings, may be a limitation for this study. Also, this pilot study did not include parameters such as psychological stress, physical activity, dietary factors, chronic illnesses, medications, hormonal contraceptive use, previous menstrual characteristics, COVID-19 severity, fever, interval since infection, vaccine type, and number of vaccine doses, which may be considered as confounders, comprising another major limitation. Finally, the Pictorial Blood Assessment Chart (PBAC) was not employed, and this may be considered as a limitation.

On the other hand, this study has several strengths. To our knowledge, this is the first Greek study to assess the risk of menstrual disorders after SARS-CoV-2 exposure in adolescent girls, addressing a gap, since most research focuses on adult women. An additional strength of the study is its comprehensive laboratory analysis of the adolescents, including blood, biochemical, and hormonal tests, to identify potential causes of menstrual disorders. This detailed evaluation revealed differences between adolescents with COVID-19, those vaccinated, and those unexposed, providing insights into risk factors and the link between exposure to SARS-CoV-2 and menstrual irregularities. The results can help healthcare professionals improve management and provide targeted guidance for prevention and monitoring. Lastly, this study lays the foundation for future research on the long-term effects of COVID-19 and vaccination against SARS-CoV-2 on adolescent reproductive health. Its findings may prompt larger, multicenter studies across diverse populations, ultimately guiding policies to improve healthcare and awareness globally.

Menstrual disorders are a prevalent concern among adolescent girls and can be influenced by a variety of factors. Emerging evidence suggests that exposure to SARS-CoV-2—whether via infection or vaccination—may contribute to alterations in the menstrual cycle patterns. Considering these findings, growing parental concern regarding the potential impact of COVID-19 and its vaccines on adolescent development and future fertility has led to increased vaccine hesitancy. It is; therefore, imperative that healthcare professionals are well-informed about the possible associations between SARS-CoV-2 exposure and menstrual irregularities in adolescent populations. Recognizing that these abnormalities are not uncommon is essential in providing appropriate counseling, mitigating unwarranted fears and supporting informed decision making among families.

To conclude, the findings of this study suggest that SARS-CoV-2 exposure, either through vaccination or infection, may impact the endocrine and hemostatic systems, potentially contributing to menstrual disorders in adolescent girls. Further research is essential to investigate the underlying pathophysiological mechanisms in this understudied age group. Larger samples of adolescents are needed to confirm our results and draw more reliable conclusions. Long-term follow-up is also essential to determine whether such menstrual changes are temporary or if they may affect future reproductive health. Lastly, future studies should assess the risk of recurrence following subsequent SARS-CoV-2 exposure. A deeper understanding of these effects will improve clinical care and strengthen public trust in medical science. 

## Figures and Tables

**Table 1:** Differences in hematological parameters between SARS-CoV-2 and control groups.

Variables	Control Group (*N* = 15)	SARS-CoV-2 Group (*N* = 77)	*p*-Value	Lower 95% CI	Upper 95% CI
Hb (g/dL)	12.02	11.9	0.788	−0.78	1.02
Hct (%)	37.05	36.43	0.585	−1.66	2.88
WBC (10^3^/μL)	6.48	7	0.232	−1.39	0.35
PLT (10^3^/μL)	285.07	289.47	0.773	−35.52	26.71
Glu (mg/dL)	87.27	88.06	0.73	−5.53	3.95
Ins (μUI/mL)	16.72	14.06	0.549	−7.07	12.39
HbA1c (%)	4.95	5.2	0.175	−0.64	0.14
Urea (mg/dL)	29.57	26.92	0.369	−3.43	8.72
Creat (mg/dL)	0.67	0.7	0.6	−0.18	0.1
Uric Acid (mg/dL)	4.28	4.03	0.479	−0.49	0.99
Chol (mg/dL)	155.78	157.27	0.925	−35.47	32.49
TG (mg/dL)	76.15	78.9	0.82	−27.95	22.44
HDL (mg/dL)	55.32	55.64	0.922	−7.33	6.68
LDL (mg/dL)	80.55	89.37	0.486	−35.35	17.7
SGOT (U/L)	15.83	18.09	0.097	−4.96	0.44
SGPT (U/L)	13.91	14.99	0.671	−6.35	4.2
γ-GT (U/L)	10.09	13.33	0.06	−6.63	0.14
ALP (U/L)	128.35	103	0.262	−21.38	72.09
ALB (g/dL)	4.61	4.62	0.958	−0.37	0.35
CPK (U/L)	60.5	91.19	0.065	−63.63	2.23
Fe (μg/dL)	88.33	63.27	0.056	−0.74	50.85
Ferritin (μg/dL)	39.38	35.27	0.775	−26.01	34.22
B12 (pg/mL)	627.5	421.55	0.402	−1504.77	1916.66
25OHD3 (ng/mL)	21.83	21.45	0.913	−6.68	7.44
Folic acid (ng/mL)	6	8.77	0.666	−16.22	10.67
TSH (μU/mL)	3	2.07	0.459	−1.73	3.59
FT4 (ng/dL)	2.02	3.56	0.391	−5.14	2.04
PTH (pg/mL)	50.58	46.83	0.711	−17.87	25.37
FSH (mIU/mL)	7.62	5.81	0.474	−3.56	4.2
LH (mIU/mL)	6.01	8.21	0.165	−5.38	0.98
E2 (pg/mL) *	27.67	53.2	<0.001	−38.67	−12.39
PRL (ng/mL)	19.47	15.62	0.259	−3.17	10.85
17-OHP (ng/mL)	1.35	2.02	0.159	−1.62	0.27
D4 (ng/mL)	2.67	1.96	0.071	−0.68	1.47
DHEAS (μg/dL)	179.51	200.61	0.468	−80.78	38.59
Testo (ng/dL)	15.35	11.66	0.514	−8.07	15.45
SHBG (nmol/L)	50.26	49.74	0.95	−16.07	17.1
Cort (ng/mL)	17.9	61.43	0.19	−116.02	28.95
Na (mmol/L)	139.53	136.52	0.287	−2.61	8.62
K (mmol/L)	4.53	4.31	0.411	−0.38	0.8
Ca (mg/dL)	9.89	9.67	0.218	−0.14	0.57
P (mg/dL)	4.41	4.25	0.339	−0.19	0.52
Mg (mg/dL)	2.06	2.01	0.279	−0.64	0.16
PT (s)	12.46	12.88	0.526	−1.88	1.03
INR	1.12	1.12	0.956	−0.13	0.12
APTT (s)	29.71	29.92	0.847	−2.72	2.27
Fibrinogen (mg%)	292.85	267.17	0.296	−27.18	78.52
VIII (%)	116.66	119.92	0.828	−37.14	30.62
vWFAg (%)	94.95	95.6	0.98	−68.75	67.44
Rcof (%)	113.66	92.76	0.105	−4.93	46.75

* *p* < 0.001. *Hemoglobin (Hb), hematocrit (Hct), white blood cell (WBC), platelet (PLT), glucose (Glu), insulin (Ins), hemoglobin A1c (HbA1c), urea (Urea), creatinine (Creat), cholesterol (Chol), triglycerides (TG), high-density lipoprotein (HDL), low-density lipoprotein (LDL), serum glutamic-oxaloacetic transaminase (SGOT), serum glutamic pyruvic transaminase (SGPT), gamma-glutamyl transferase (γ-GT), alkaline phosphatase (ALP), albumin (ALB), creatine phosphokinase (CPK), iron (Fe), vitamin B12 (B12), 25-hydroxyvitamin D3 (25OHD3), thyroid-stimulating hormone (TSH), free thyroxine (FT4), parathyroid hormone (PTH), follicle-stimulating hormone (FSH), luteinizing hormone (LH), estradiol (E2), prolactin (PRL), 17-hydroxyprogesterone (17-OHP), delta-4-androstenedione (D4), dehydroepiandrosterone sulfate (DHEAS), testosterone (Testo), sex hormone-binding globulin (SHBG), cortisol (Cort), sodium (Na), potassium (K), calcium (Ca), phosphorus (P), magnesium (Mg), prothrombin time (PT), international normalized ratio (INR), activated partial thromboplastin time (APTT), factor VIII (VIII), von Willebrand factor antigen (vWFAg), and ristocetin cofactor (Rcof).*

**Table 2:** Differences in hematological parameters between vaccinated and unvaccinated adolescents.

Variables	Unvaccinated Group	Vaccinated Group	*p*-Value	Lower 95% CI	Upper 95% CI
Hb (g/dL)	11.9	11.96	0.86	−0.81	0.68
Hct (%)	36.47	36.63	0.882	−2.27	1.95
WBC (10^3^/μL)	6.68	7.26	0.149	−1.37	0.21
PLT (10^3^/μL)	292.97	282.94	0.495	−19.05	39.13
Glu (mg/dL)	89.1	86.21	0.089	−0.45	6.24
Ins (μUI/mL)	14.94	13.98	0.707	−4.15	6.07
HbA1c (%)	5.11	5.23	0.62	−0.35	0.11
Urea (mg/dL)	27.03	27.82	0.717	−5.14	3.56
Creat (mg/dL)	0.62	0.81	0.147	−4.51	0.07
Uric Acid (mg/dL)	4.01	4.16	0.708	−0.94	0.65
Chol (mg/dL)	164.73	146.22	0.054	−0.31	37.33
TG (mg/dL)	78.25	78.8	0.96	−22.57	21.45
HDL (mg/dL)	55.75	55.36	0.887	−5.09	5.87
LDL (mg/dL)	94.78	78.5	0.07	−1.38	33.94
SGOT (U/L)	17.39	18.24	0.52	−3.47	1.78
SGPT (U/L)	15.07	14.45	0.743	−3.15	4.4
γ-GT (U/L)	12.18	13.98	0.543	7.76	4.17
ALP (U/L)	106.16	113.31	0.639	−37.84	23.54
ALB (g/dL)	4.59	4.66	0.395	−0.25	0.1
CPK (U/L)	93.33	83.09	0.668	−38.83	59.31
Fe (μg/dL)	69.67	64.08	0.705	−24.51	32.69
Ferritin (μg/dL)	27.6	46.76	0.068	−39.74	1.43
B12 (pg/mL)	421.35	473.37	0.169	−163.17	59.13
25OHD3 (ng/mL)	19.37	24.23	0.343	−15.18	5.46
Folic acid (ng/mL)	8.59	8.64	0.986	−5.85	5.75
TSH (μU/mL)	2.36	2.03	0.408	−0.45	1.11
FT4 (ng/dL)	2.05	4.78	0.307	−8.07	2.62
PTH (pg/mL)	44.07	53.68	0.266	−27.1	7.87
FSH (mIU/mL)	6.59	5.54	0.219	−0.64	2.75
LH (mIU/mL)	8.88	6.62	0.105	−0.48	4.99
E2 (pg/mL)	49.95	48.18	0.847	−16.41	19.94
PRL (ng/mL)	17.61	14.5	0.103	−0.65	6.87
17-OHP (ng/mL)	1.39	2.49	0.151	−0.26	0.42
D4 (ng/mL)	2.19	2.01	0.507	−0.36	0.73
DHEAS (μg/dL)	211.56	177.9	0.163	−13.93	81.25
Testo (ng/dL)	10.8	13.99	0.428	−10.96	4.7
SHBG (nmol/L)	47.32	53.2	0.596	−28.28	16.51
Cort (ng/mL)	44.41	49.43	0.911	−102.75	92.72
Na (mmol/L)	139.29	132.13	0.329	−7.97	22.29
K (mmol/L)	4.36	4.3	0.55	−0.15	0.29
Ca (mg/dL)	9.75	9.63	0.252	−0.08	0.33
P (mg/dL)	4.24	4.32	0.598	−0.36	0.21
Mg (mg/dL)	2.03	1.99	0.329	−0.05	0.14
PT (s)	12.82	12.84	0.977	−1.08	1.05
INR	1.14	1.1	0.336	−0.04	0.11
APTT (s)	29	31.2	0.122	−5.03	0.63
Fibrinogen (mg%)	263.56	283.46	0.33	−60.94	21.14
VIII (%)	136.25	86.3	0.002	20.39	79.5
vWFAg (%)	108.54	71.84	0.023	5.38	68
Rcof (%)	110.83	65.5	0.007	13.14	75.52

*Hemoglobin (Hb), hematocrit (Hct), white blood cell (WBC), platelet (PLT), glucose (Glu), insulin (Ins), hemoglobin A1c (HbA1c), urea (Urea), creatinine (Creat), cholesterol (Chol), triglycerides (TG), high-density lipoprotein (HDL), low-density lipoprotein (LDL), serum glutamic-oxaloacetic transaminase (SGOT), serum glutamic pyruvic transaminase (SGPT), gamma-glutamyl transferase (γ-GT), alkaline phosphatase (ALP), albumin (ALB), creatine phosphokinase (CPK), iron (Fe), vitamin B12 (B12), 25-hydroxyvitamin D3 (25OHD3), thyroid-stimulating hormone (TSH), free thyroxine (FT4), parathyroid hormone (PTH), follicle-stimulating hormone (FSH), luteinizing hormone (LH), estradiol (E2), prolactin (PRL), 17-hydroxyprogesterone (17-OHP), delta-4-androstenedione (D4), dehydroepiandrosterone sulfate (DHEAS), testosterone (Testo), sex hormone-binding globulin (SHBG), cortisol (Cort), sodium (Na), potassium (K), calcium (Ca), phosphorus (P), magnesium (Mg), prothrombin time (PT), international normalized ratio (INR), activated partial thromboplastin time (APTT), factor VIII (VIII), von Willebrand factor antigen (vWFAg), and ristocetin cofactor (Rcof).*

**Table 3:** Differences in hematological parameters between COVID-19 infected and non-infected groups.

Variables	Non-Infected Group (Mean Value)	Infected Group (Mean Value)	*p*-Value	Lower 95% CI	Upper 95% CI
Hb (g/dL)	12.29	11.8	0.209	−0.28	1.27
Hct (%)	37.79	36.09	0.093	−0.29	3.68
WBC (10^3^/μL)	6.66	7.01	0.363	−1.13	0.42
PLT (10^3^/μL)	281.23	291.31	0.496	−39.62	19.46
Glu (mg/dL)	87.6	88.05	0.823	−4.44	3.56
Ins (μUI/mL)	16.32	14.02	0.528	−5.4	10.01
HbA1c (%)	4.96	5.22	0.076	−0.53	0.03
Urea (mg/dL)	28.4	26.97	0.505	−2.86	5.71
Creat (mg/dL)	0.86	0.64	0.244	−0.15	0.59
Uric Acid (mg/dL)	4.13	1.04	0.828	−0.81	0.99
Chol (mg/dL)	156.73	157.14	0.973	−24.76	23.95
TG (mg/dL)	83.09	76.93	0.641	−20.66	32.97
HDL (mg/dL)	54.36	56.01	0.519	−6.81	3.49
LDL (mg/dL)	84.15	89.27	0.606	−25.09	14.85
SGOT (U/L)	17.55	17.8	0.882	−3.67	3.18
SGPT (U/L)	14.83	14.82	0.97	−5.05	5.07
γ-GT (U/L)	10.04	13.86	0.028 *	−7.19	−0.43
ALP (U/L)	124.8	101.05	0.168	−10.69	58.19
ALB (g/dL)	4.62	4.61	0.906	−0.21	0.24
CPK (U/L)	73.16	93.79	0.402	−72.41	31.15
Fe (μg/dL)	88	62.43	0.033 *	2.21	48.91
Ferritin (μg/dL)	48.95	31.88	0.193	−9.26	43.4
B12 (pg/mL)	538.2	412.1	0.144	−55.45	299.65
25OHD3 (ng/mL)	21.1	21.67	0.867	−7.38	6.24
Folic acid (ng/mL)	8.54	8.63	0.974	−5.75	5.58
TSH (μU/mL)	2.7	2.04	0.988	−0.89	2.21
FT4 (ng/dL)	2.25	3.7	0.435	−5.12	2.23
PTH (pg/mL)	49.1	47.06	0.812	−15.61	19.69
FSH (mIU/mL)	6.83	5.83	0.516	−2.13	4.11
LH (mIU/mL)	6.85	8.23	0.309	−4.04	3.3
E2 (pg/mL)	38.18	53.29	0.057	−30.66	0.44
PRL (ng/mL)	16.6	16.24	0.89	−4.93	5.65
17-OHP (ng/mL)	1.34	2.12	0.123	−1.79	0.22
D4 (ng/mL)	2.4	1.96	0.141	−0.15	1.03
DHEAS (μg/dL)	169.08	207.15	0.121	−86.58	10.44
Testo (ng/dL)	14.61	11.33	0.459	−5.61	12.16
SHBG (nmol/L)	44.92	52.22	0.394	−24.38	9.77
Cort (ng/mL)	19.55	66.47	0.228	−132.32	38.49
Na (mmol/L)	139.52	136.03	0.28	−2.97	9.95
K (mmol/L)	4.42	4.32	0.49	−0.2	0.4
Ca (mg/dL)	9.74	9.69	0.695	−0.2	0.29
P (mg/dL)	4.41	4.22	0.196	−0.1	0.47
Mg (mg/dL)	2.03	2.02	0.729	−0.04	0.06
PT (s)	12.48	12.94	0.33	−1.42	0.491
INR	1.11	1.13	0.707	−0.09	0.06
APTT (s)	30.88	29.54	0.351	−1.57	4.24
Fibrinogen (mg%)	312.31	257.26	0.023 *	8.55	101.53
VIII (%)	108	121.92	0.364	−45.62	17.78
vWFAg (%)	88.8	97.13	0.661	−49.57	32.9
Rcof (%)	102.2	93.43	0.532	−20.12	37.65

*Hemoglobin (Hb), hematocrit (Hct), white blood cell (WBC), platelet (PLT), glucose (Glu), insulin (Ins), hemoglobin A1c (HbA1c), urea (Urea), creatinine (Creat), cholesterol (Chol), triglycerides (TG), high-density lipoprotein (HDL), low-density lipoprotein (LDL), serum glutamic-oxaloacetic transaminase (SGOT), serum glutamic pyruvic transaminase (SGPT), gamma-glutamyl transferase (γ-GT), alkaline phosphatase (ALP), albumin (ALB), creatine phosphokinase (CPK), iron (Fe), vitamin B12 (B12), 25-hydroxyvitamin D3 (25OHD3), thyroid-stimulating hormone (TSH), free thyroxine (FT4), parathyroid hormone (PTH), follicle-stimulating hormone (FSH), luteinizing hormone (LH), estradiol (E2), prolactin (PRL), 17-hydroxyprogesterone (17-OHP), delta-4-androstenedione (D4), dehydroepiandrosterone sulfate (DHEAS), testosterone (Testo), sex hormone-binding globulin (SHBG), cortisol (Cort), sodium (Na), potassium (K), calcium (Ca), phosphorus (P), magnesium (Mg), prothrombin time (PT), international normalized ratio (INR), activated partial thromboplastin time (APTT), factor VIII (VIII), von Willebrand factor antigen (vWFAg), and ristocetin cofactor (Rcof).* * statistical significance.

## Data Availability

The data presented in this study are available on request from the corresponding author.
